# Spontaneous Purification of Thames Water

**Published:** 1830-04-01

**Authors:** 


### Spontaneous Purification of Thames Water.

Dr. Bostock has published a paper on this subject in the Philosophical Transactions, which may afford some consolation to the involuntary drinkers of Thames puddle and common sewer scourings—namely, that, if they can keep the water in reservoirs for some weeks, the contents of privies with which it is so fully imbued, will be converted into very comfortable and wholesome medicines, viz lime, sulphuric acid, magnesia, &c. Dr. Bostock was supplied with a certain quantity of water, "taken in the river, in the current of, and immediately at the mouth of the King's Scholars' pond sewer." He described it to the Commissioners as "in a state of extreme impurity, opaque with filth, and exhaling a highly fetid odour." It was, after a week, passed through a layer of sand and charcoal—and then suffered to rest for several weeks, when it was found that a great change had taken place in its appearance. It had become much clearer, while nearly the whole of the sediment had risen to the surface, forming a pretty regular stratum of about *half an inch in thickness*—the odour still continuing extremely offensive—"perhaps even more so than at first." In eight weeks more, the water became perfectly transparent, without any unpleasant odour, though still retaining somewhat of its original dingy colour. The scum subsided in masses to the bottom leaving the water apparently free from extraneous matters. It was filtered through paper and analyzed—when it was found to contain carbonate of lime—sulphate of ditto, muriate of soda, and muriate of magnesia. "The results of this analysis," says the Doctor, "show that, although the water has, by this depurating process, freed itself from the great quantity of organic matter which it contained, and acquired a state of *apparent* purity, which might render it sufficiently proper for many purposes (not we should think for drinking) yet that the quantity of saline matter is increased fourfold." This depurating process, Dr. B. considers a species of fermentation, an operation, wherein a substance, without any addition, undergoes a change in the arrangement of its component parts, and a new compound or compounds are produced. The source of the saline bodies abovementioned, he supposes to be the organic substances, "principally of an animal origin, which are so copiously deposited in the Thames water:—Of these the most abundant are the *excrementitious matters*, as well as the parts of various undecomposed animal bodies."

"The different species of the softer and more soluble animal compounds act as the ferment, and are themselves destroyed, while the salts which are attached to them are left behind. It may be conceived therefore that the more foul the water is, the more complete will be the subsequent process of depuration; and we have hence an explanation of the popular opinion, that the Thames water is peculiarly valuable for sea stores, its extreme impurity inducing the fermentative process, and thus removing from it all those substances which can cause it to undergo any further alteration."

It is to be remembered that Thames water for shipping is generally taken up about Greenwich, or even so low down as Woolwich—at least this was the place from whence water was taken up, and which we had the misery of drinking all the way to China—holding our noses at the same time, as the smell was infinitely worse than the taste. It is very true that, after many months standing, or rather rolling in the holds of ship, this water becomes clear, and comparatively free from disagreeable odour—but no chemistry, we imagine, can reconcile us to this change of excrementitious matters into carbonates, sulphates, muriates, and other names however familiar to our pharmacopœial ears! It is astonishing, considering that three fourths of our senators are obliged to drink this excrementitious fluid during their parliamentary duties in London, that they do not rise up, one and all, to stop the evil by laws of which they are the framers!

Dr. Bostock's paper is written with such obscurity and confusion that we had some difficulty in making out its true sense and bearings.

